# Revealing cytotoxic substructures in molecules using deep learning

**DOI:** 10.1007/s10822-020-00310-4

**Published:** 2020-04-16

**Authors:** Henry E. Webel, Talia B. Kimber, Silke Radetzki, Martin Neuenschwander, Marc Nazaré, Andrea Volkamer

**Affiliations:** 1 In silico Toxicology and Structural Bioinformatics, Institute of Physiology, Charité-Universitätsmedizin Berlin, Charitéplatz 1, 10117 Berlin, Germany; 2 Leibniz-Forschungsinstitut für Molekulare Pharmakologie (FMP), Robert-Roessle Strasse 10, 13125 Berlin, Germany

**Keywords:** Deep Neural Networks, Deep Taylor Decomposition, Cytotoxic substructures, Toxicophores

## Abstract

In drug development, late stage toxicity issues of a compound are the main cause of failure in clinical trials. In silico methods are therefore of high importance to guide the early design process to reduce time, costs and animal testing. Technical advances and the ever growing amount of available toxicity data enabled machine learning, especially neural networks, to impact the field of predictive toxicology. In this study, cytotoxicity prediction, one of the earliest handles in drug discovery, is investigated using a deep learning approach trained on a highly consistent in-house data set of over 34,000 compounds with a share of less than 5% of cytotoxic molecules. The model reached a balanced accuracy of over 70%, similar to previously reported studies using Random Forest. Albeit yielding good results, neural networks are often described as a black box lacking deeper mechanistic understanding of the underlying model. To overcome this absence of interpretability, a Deep Taylor Decomposition method is investigated to identify substructures that may be responsible for the cytotoxic effects, the so-called toxicophores. Furthermore, this study introduces cytotoxicity maps which provide a visual structural interpretation of the relevance of these substructures. Using this approach could be helpful in drug development to predict the potential toxicity of a compound as well as to generate new insights into the toxic mechanism. Moreover, it could also help to de-risk and optimize compounds.

**Electronic supplementary material:**

The online version of this article (10.1007/s10822-020-00310-4) contains supplementary material, which is available to authorized users.

## Introduction

Over the past two decades, an increasing number of new chemicals have been synthesized every year [1] and fast prior analysis of their potentially toxic effects on humans and animals has become crucial [2]. In drug development, late stage safety and toxicity issues are still the main causes of failure in clinical trials [3, 4]. Moreover many animals (ca. 2.8 Mio, BMEL [5]) are deployed for testing in research and development. Therefore, in silico methods are highly valuable during early drug development to reduce costs, human discomfort and animal testing [6] and might contribute to the early identification of harmful substances according to the REACH regulation [7]. *Machine learning (ML)* algorithms, more specifically deep learning methods, have proven to perform well in different fields, such as speech recognition [8] or image classification [9], and are now also broadly used in drug design [10–14]. A recent review of deep learning in chemistry can be found in [15]. ML-based endpoint prediction in computational chemistry follows the principle that compounds with similar substructures or features may cause similar effects. Given a labeled data set with known outcome, the ML algorithm learns to identify the often highly non-linear combination of physico-chemical and structural features in the compound, commonly encoded by circular fingerprints (e.g. Morgan/ECFP), that may be responsible for their (toxic) effect [16–19]. Such models can be built for target-specific endpoints (binding assays) as well as for more complex biological endpoints (cell-based assays), such as cytotoxicity. While more data might be available for the former group, the models might be less relevant for in vivo situations [20].

Cellular *cytotoxicity* is a high-level property of molecules as it can be caused by different mechanisms. It refers to cell-death by cell membrane damage and necrotic lysis or cell processes such as apoptosis, autophagy or regulated necrosis [21]. Cytotoxicity is experimentally assessed by counting survival rates after treating a cell line with a given substance [22]. In pharmaceutical drug discovery, cytotoxicity is one of the earliest handles for assessing toxicity of a drug. Discarding compounds with undesired features early in the development stage is of high practical value, following the “fail early - fail cheap” de-risking principle.

Some *computational cytotoxicity* models have already been published, most of them applying random forest algorithms [21, 23, 24], others using Bayesian methods with physico-chemical properties and/or circular fingerprints as descriptors [25]. Additionally, a naive Bayes approach in combination with activity spectra has been introduced for cytotoxicity prediction [26]. Furthermore, previous studies have shown the success of *Feedforward Neural Networks (FNN)* [27, Ch.6] especially in predicting different toxic endpoints [28, 29]. The ability of such networks to model and learn non-linear, complex relationships have gained more and more attention in the context of chemistry [30]. While showing promising results, two major challenges remain for such methods in drug design.

The first challenge is the availability of sufficient and reliable data [31]. Many models are trained on scattered publicly available - and thus, heterogeneous data - due to assay diversity, as well as highly variable conditions and setups used throughout different laboratories. Therefore, thorough data curation is crucial [32]. Second, ML algorithms and especially Deep Neural Networks (DNN) may act as a black box and one is often unable to understand the intricacies in the hidden layers. The deeper the network the more complicated the interpretation becomes. Over the last years, several techniques to interpret such models have been introduced in the broader context of drug discovery [33–38], including but not limited to atom-level coloration [34], integrated gradients [35], attention-vector based relevant latent features exploration [36], masking and gradient techniques applied to 3D convolutional neural networks [37] and partial derivative-based methods [38].

To overcome these hurdles, a DNN model is trained in this study using a highly consistent data set from the Leibniz Associations Research Institute for Molecular Pharmacology (FMP: Leibniz-Forschungsinstitut für Molekulare Pharmakologie), with approximately 34,000 compounds (remaining standardized compounds after data preprocessing) measured for their cytotoxic potential. The effect on cell viability, including sublethal effects on cell proliferation, was measured using a high-content screening assay. This assay enables to visualize and quantify phenotypic changes due to compound treatment. Furthermore, a new technique is used here to unleash the black box effect by identifying relevant features for toxicity prediction. One recent approach, known as the layer-wise relevance propagation (LRP), decomposes the output scores layer by layer back to the original inputs of the network, yielding information on which features are important for the prediction. One special case of the LRP method, called *Deep Taylor Decomposition (DTD)* developed by Montavon et al.[39], uses the Taylor decomposition to redistribute the output score. This study is the first, to the best of our knowledge, that uses the DTD in the molecular context. In order to obtain a visual representation of the atom environments potentially relevant for cytotoxicity determined by the DTD method, a technique developed by Riniker and Landrum [40], called similarity maps, is employed to depict the 2D plots of the molecules where the relevances of the potentially cytotoxic substructures are highlighted. The application of similarity maps in the context of cytotoxicity prediction will further be referred to as cytotoxicity maps. With this approach, potential cytotoxic compounds could be identified and prioritized for experimental testing and verification.

## Data and methods

This section describes the data set and the preprocessing steps, as well as the machine learning models that are used for this study. Furthermore, the Deep Taylor Decomposition to identify potential toxicophores and the visualization using cytotoxicity maps are introduced.

### Data

#### Data collection and cytotoxicity definition

The compound library available at the FMP comprises a collection of 74,000 chemically distinct substances that were assembled at the FMP [41]. Among them, more than 34,000 compounds were purchased from commercial vendors. These commercial compounds were selected after an analysis of the World Drug Index (database of 70,000 approved drugs and natural products annotated for bioactivity) for privileged substructures frequently occurring in different drugs. According to the approximately 561 identified main chemotypes, which represent a major part of the currently known chemical space of drug-like molecules, compounds presenting these privileged motifs in different combinations and variations were selected. Prior incorporation into the library, a filtering against known reactive groups (similar to filtering against pan-assay interference compounds [42]) was performed as described in Lisurek et al. [41].

The initial data set from the FMP available for this study contained 34,848 compounds that were tested for their cytotoxic effects on two cell lines, HepG2 and HEK293, as well as another 1408 compounds that were tested only on the HepG2 cell line. Cells were seeded onto 384-well plates, compounds added to a concentration of 10 *μ*M, and cells incubated for additional 72 hours. Resulting cell numbers were then determined by staining of the nuclei using Hoechst 33342 technique^1^ [43] and counting the nuclei with fluorescence microscopy. In order to increase reliability, three technical replicates (replicating the steps of cell seeding, compound addition and cell counting) were generated. The high concentration justifies two assumptions: first, the permeability of molecules does not need to be taken into account as the high concentration likely leads to cell membrane penetration and relevant intracellular concentrations. Second, the high concentration should also reliably reveal existing toxicity of the compounds.

Cytotoxicity of a molecule is defined using the relative growth inhibition measurement comparing two samples of a cell line, untreated and treated, respectively. A molecule is labeled cytotoxic if it inhibits growth by at least 50% compared to the untreated samples and the cell count should be three standard deviations lower than the median of the cell lines on a specific plate. This effect had to be observed in at least two of the three technical replicates.

In case a compound is toxic at the same concentration range as applied for the measurements $(10\, \mu \hbox {M})$, small differences in sensitivity between the different cell lines may lead to a compound being determined toxic in one cell line but not in the other. Thus for this study, a compound is considered cytotoxic if it is measured cytotoxic on at least one of the two cell lines (HEK293 or HepG2).

#### Compound data preprocessing

**Fig. 1:**
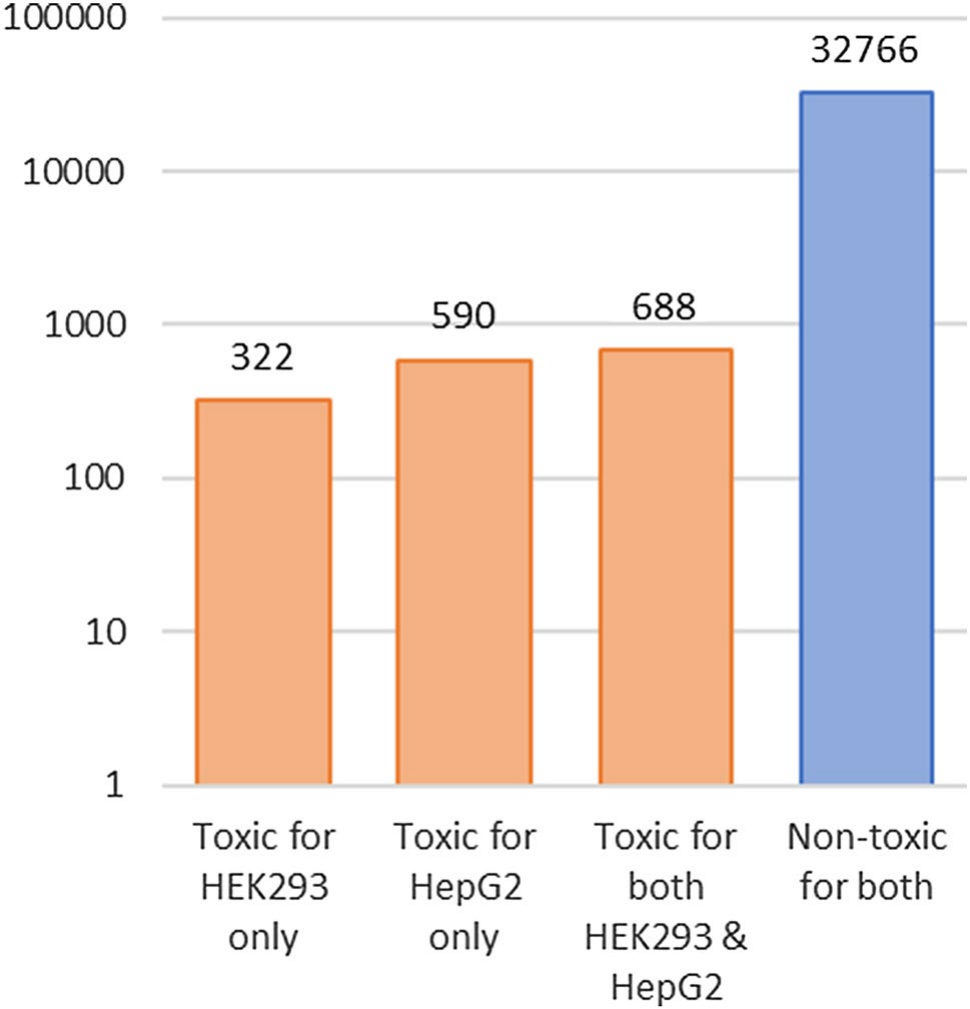
The logarithmic scale plot shows the number of toxic and non-toxic molecules for the two cell lines HEK293 and HepG2. There are approximately 20 times more molecules that are labeled non-toxic than toxic, making the data set highly imbalanced

All molecules are processed with RDKit [44], of which 157 are discarded due to sanitization issues. After sanitization, the remaining molecules are preprocessed by applying certain structure standardization rules, e.g. removing salts, normalizing charges and handling tautomers, using the tool developed in the scope of IMI eTox [45]. Subsequently, duplicates produced by the standardization process are removed. This results in 34,366 compounds that are considered in this study. Only 4.65% of the molecules in the preprocessed data set are labeled cytotoxic, leading to highly imbalanced data (see Fig. 1).

#### Compound encoding

All molecules in the preprocessed data set are transformed into Morgan fingerprints using RDKit [44]. Atom environments are only considered at an exact radius of two bonds and the length of the fingerprint is set to 2048. Environments are only included if they appear at least five times in the data set, yielding 14,245 unique hash keys. This selection omits 40,507 substructures as they were present less than five times in the data set. This feature selection is equivalent to the first step of Gütlein and Kramer [46, Table 6]. Note that due to the hashing of the features to a 2048 bit fingerprint, different atom environments may be mapped to the same bit, known as bit collision.

### Machine learning model generation

#### FNN model setup

In this study, a feedforward fully-connected neural network (FNN) is used to predict cytotoxicity of compounds, a model similar to Gütlein and Kramer [33] in the TOX21 challenge. The inputs are given by the 2048 long fingerprints and the outputs are binary variables indicating if a molecule is cytotoxic or not. The architecture of the model considers three dense hidden layers with respectively 512, 192 and 128 units. The activation function used in the hidden layers of the network is the ReLU function, defined as $\text {ReLU}(x)= \max \{x, 0\}$ [27, p.170]. For the final classification, a sigmoid function, defined as $\sigma (x)=\frac{1}{1+e^{-x}}$, is applied to obtain prediction values that range between 0 and 1. These values correspond to the probability of belonging to either the cytotoxic or the non-cytotoxic class. To avoid overfitting, the output layer is regularized using dropout [47], where 40% of hidden units in the last hidden layer are set to zero at random during each mini-batch gradient updating step. Additionally, toxic molecules are weighted five times more in the loss function than non-toxic ones in order to statistically increase their prevalence. The Adam method [48] is chosen as the network optimizer with an initial learning rate of 0.0001. The model has been established by running a random hyperparameter search (data not shown).

#### RF baseline model setup

To compare the results of the deep learning model, a baseline is computed using a Random Forest (RF) model. This tree-based method has shown to perform particularly well in cheminformatics [49]. The default settings in Scikit-learn [50] are used; more specifically 50 trees are fitted, each of them selecting randomly 45 out of the 2048 bits of the fingerprint as features. The same strategy as for FNN is used to account for the imbalanced data.

#### Model validation

As a model setup, a 10-fold nested cross-validation with validation and test set is used. The preprocessed data is randomly split into 10 parts. First, one of these parts is randomly selected as test set (10% of the data set), another as validation set (10% of the data) and the remaining as training set (80% of the data). Finally, all possible combination of these three sets are considered leading to 90 model evaluations (see Table 1). For each combination, also called run, the FNN and the RF models as previously described are trained on the training set, using the validation set for hyperparameter tuning, and evaluated on the test set. Note that for the FNN production run and the toxicophore evaluation, a separate model with a random split into the same proportions has been setup.

For model evaluation, the balanced accuracy (AccB) [51], the true positive rate (TPR) and the true negative rate (TNR) [52, Table1] are used as comparison metrics. The formulas for these three metrics are shown in Eqs. 1, 2 and 3, where TP represents the true positive counts, TN the true negative counts, FP the false positive counts and FN the false negative counts. Note that AUC values are not included since this metric may be misleading when evaluating model performance on imbalanced data sets, as suggested by Saito and Rehmsmeier [52].

1 $$\begin{aligned} \text{AccB}= & {} \frac{1}{2} \Big ( \text{TPR} + \text{TNR}\Big ), \end{aligned}$$

2 $$\begin{aligned} \text{TPR}= & {} \frac{ \text{TP}}{ \text{TP + FN}}, \end{aligned}$$

3 $$\begin{aligned} \text{TNR}= & {} \frac{ \text{TN}}{ \text{TN + FP}}. \end{aligned}$$

**Table 1:** Number of toxic and non-toxic compounds in each of the split sets: training, validation and test

	Training (80%)	Validation (10%)	Test (10%)	Total (100%)
Non-toxic compounds	26,212	3277	3277	32,766
Toxic compounds	1280	160	160	1600
Total compounds	27,492	3437	3437	34,366

### Deep Taylor Decomposition

When training a model, besides model performance, the relevance of certain features that lead to the predictions may be of high interest. For this purpose, Bach et al. [53] proposed a method to decompose layer-wise a given model score and redistribute the decomposed scores to the inputs. For a specific input $x$, node *i* and layer $l=0,\dots ,L$, we note $R_i^l (x)$ the associated relevance score. The layer-wise relevance propagation has the desired property to redistribute the overall relevance between two layers, meaning that the sum over the relevances assigned to the inputs equals the probability of the model score. The initial relevance, $R^L (x)$, is given by the model score.

The relevance is back-propagated to previous layers following only positive weights. This is known as the $z^+$ rule. Let $w_{ij}= w_{ij}^{l,l+1}$ be the weight that connects non-zero hidden node $x_i$ in layer *l* with hidden node $x_j$ in layer $l+1$. Only positive weights are considered, namely $w_{ij}^+= \max (0,w_{ij})$. Then the $z^+$ rule is defined as follows

4 $$\begin{aligned} R_i^{l} = \sum _j \frac{x_i^l w_{ij}^+}{\sum _k x_k^l w_{kj}^+} R_j^{l+1} = \sum _j \frac{z_{ij}^+}{\sum _k z_{kj}^+} R_j^{l+1}. \end{aligned}$$

**Fig. 2:**
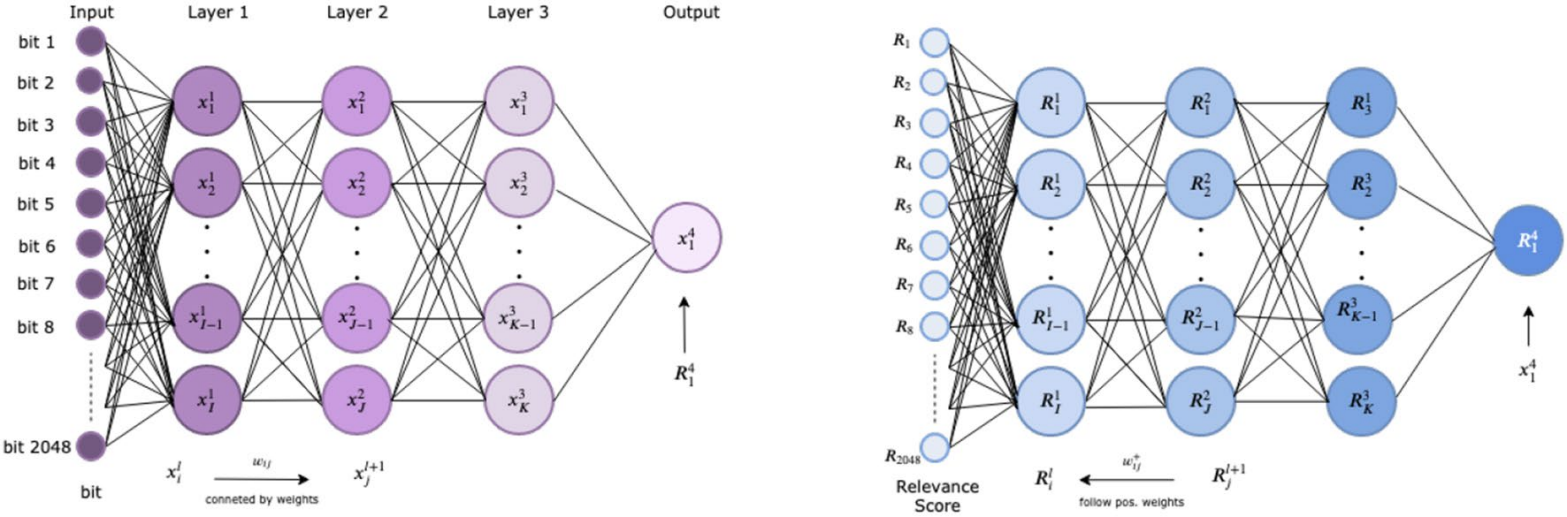
The Deep Taylor Decomposition method applied to a three hidden layer feedforward neural network. The inputs to the network are 2048 fingerprint bits. The left diagram represents the network with ReLU activation function and the right diagram the assigned relevances using the $z^+$ rule. $x^l_i, R^l_i$ represent the $i^{th}$ node, relevance at layer *l*, respectively

The name $z^+$ rule is derived from the definition $z_{ij}^+ = x_i^l w_{ij}^+$. Redistributing positive scores to the input using this rule allows to assign a positive relevance to each bit, which in this study encodes an atom environment (see Fig. 2).

Note that this method is not applied directly to the sigmoid model score, but to its logarithm of odds, $\log \big ( \frac{\sigma (x)}{1 - \sigma (x)}\big )$, the so-called logit. Model scores with positive logits, i.e. probabilities greater than 0.5, are further referred to as *decomposable*. Moreover, the method is restricting biases in ReLU activations to be negative in order to ensure the applicability of the Taylor decomposition. For further details, please refer to the paper by Montavon et al. [39].

### Identification of toxicopohores and visualization as cytotoxicity maps

To reveal the features having a high impact on the cytotoxicity classification of a molecule, the Deep Taylor Decomposition (DTD) method, as described in the previous section, is applied. Furthermore, for better interpretability, the features are mapped back to the molecular structure and are visualized using similarity maps, introducing the concept of cytotoxicity maps.

#### Detection of potential toxicophores

Toxicophores, in this study, are substructures in a molecule that highly contribute to the toxicity prediction. In order to identify the toxicophores in the data set, the bit-wise relevance scores, encoded by the fingerprint bits, are investigated and averaged over the complete set of molecules with decomposable scores. Such molecules will further be referred to as decomposable molecules.

For each decomposable molecule $m \in \{1, \ldots , M \}$ and for each fingerprint bit $j \in \{1, \ldots , N\}$, a relevance score $R_{m,j}$ is retrieved using the DTD method, see Fig. 2. The relevance scores for each bit are aggregated by taking the mean over all atom environments setting a bit in decomposable molecules, denoted as $N_j$. Therefore, each atom environment *j* will be assigned a score $R_j$ which was averaged on the selected data defined as the global mean relevance score

5 $$\begin{aligned} R_j = \frac{1}{N_j}\sum _m R_{m,j}. \end{aligned}$$

**Fig. 3:**
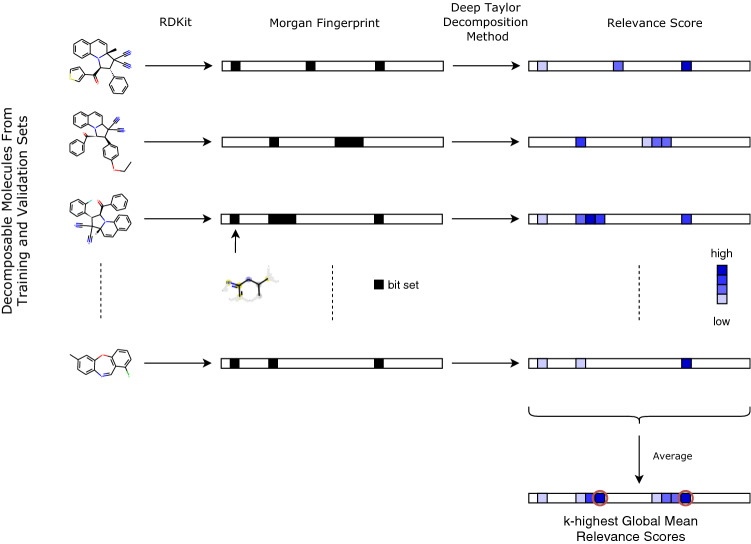
Workflow for identifying potential toxicophores. The first arrow describes the transformation from the molecules in the training and validation sets into 2048 long binary vector describing the Morgan fingerprints of radius 2, using RDKit. Each bit represents one (or more) atom environment(s). The black box indicates if the corresponding atom environment is present in the molecule. The second arrow shows that relevance scores can be obtained for each compound using the Deep Taylor Decomposition method described in the “Deep Taylor Decomposition” section and illustrated in Fig. 2. Once all relevance scores are computed for each decomposable molecule, they are averaged using Eq. 5. The bits corresponding to the *k*-highest global mean relevance scores are stored and used for further analysis as potential toxicophores

With this approach, the $k\in \mathbb {N}$ most likely cytotoxic substructures, or toxicophores, can be identified by selecting the *k* highest global mean relevance scores $R_{(1)}, \ldots , R_{(k)}$, noting $R_{(i)} \ge R_{(j)}, \, \forall i \ge j$ the ordered relevance scores. The associated workflow is illustrated in Fig. 3. For each decomposable molecule, the subset of the identified *k*-most relevant toxicophores is indicated on the structure by highlighting in red all atoms that are part of the identified relevant substructure using pre-implemented plotting functions in RDKit. If a molecule contains more than one of the most likely substructures, where these cases can include disconnected, nested or overlapping substructures, the union of these substructures is displayed (i.e. each atom that is part of at least one of these environments is highlighted once).

#### Cytotoxicity maps

To visualize the contribution of all atom environments contained in a molecule to the cytotoxicity prediction, similarity maps developed by Riniker and Landrum [40] are used. This technique allows to identify and visualize atom contribution from a prediction computed by a ML algorithm. In the original study, this is done as follows: Given a fingerprint of a molecule, a pre-trained ML model and a prediction value for the fingerprint, a set of weights for each atom in the molecule have to be calculated. These weights, which will define the atom contribution of the prediction, are computed in the following way: Recursively each atom is removed from the molecule and a new fingerprint is generated. The prediction of the new fingerprint is evaluated with the pre-trained ML model. Finally, the weight associated to that atom is the difference between the prediction of the fingerprint generated with and without the presence of that same atom. For visualization, bivariate Gaussian distributions centered at the atom position using these weights are generated and the plots show the superimposition of the atom positions and the contour lines of the distributions.

In this study, the weights are computed slightly differently. Indeed the weights considered are the relevance scores which are directly generated from the DTD method. Note that in contrast to the original work, the weights here can only be positive. However, as discussed in the “Deep Taylor Decomposition” section, theses scores are associated to each bit in a decomposable molecule and not to each atom. Therefore, the global mean relevance score is attributed to each atom in the atom environment. Consequently each atom in the decomposable molecule is mapped to a weight and the similarity map and plots can be generated in this context. Some of the substructures might overlap and have atoms in common. In this case, the weight of an atom part of several substructures will be given the maximum value of the global relevance scores associated to the atom environments. In the cytotoxicity maps, substructures with high relevance scores will stand out and could hint to toxicophores.

### Used software and libraries

RDKit [44] is used for molecular encoding, fingerprint generation and plotting of molecules. Scikit-learn [50] is employed for the Random Forest model. The deep learning model is implemented using Keras with Tensorflow backend [54]. For the score decomposition, DTD implementations as provided by iNNvestigate [55] are used. The similarity maps visualization is used as in the original paper [40].

## Results and discussion

In the following, the results of the deep learning model as well as the baseline model are discussed and then compared to other studies on in silico cytotoxicity predictions. Additionally the toxicophores identified using the DTD method and the cytoxicity maps are presented.

### Model evaluation and comparison

In this study, an FNN model for cytotoxicity prediction has been established based on the final set of 34,366 preprocessed compounds provided by the FMP, which were tested for their cytotoxic effect on two cell lines. Out of these compounds, 32,353 are commercial compounds selected using the strategy described by Lisurek et al. [41], another 2013 are commercial compounds with known biological activity (‘LOPAC®1280’ library from Sigma-Aldrich [56]) and FDA-approved drugs (‘FDA Approved Drug Library L1300’ from Selleckchem [57]). The data can be considered as highly consistent and curated, since it has been produced in the same laboratory using the same cell line and experimental setup with several reference compounds as control for each assay campaign. Note that the data set is highly imbalanced with a share of only 4.65% of toxic molecules.

#### FNN vs. RF cross-validation results

First, the results of the nested cross-validation (CV) of the FNN model are compared to the baseline RF model. Overall both the FNN and the RF models perform similarly well regarding balanced accuracy on the given data set. On the training set, RF seems to highly overfit the data (see Train row in Table 2), meaning that the model would tend to memorize patterns instead of learning them. On the test set, the FNN and RF models yield similar results with a mean balanced accuracy of approximately 68%, with a slightly higher mean and narrower standard deviation for the FNN setup (see Table 2). This is a fair increase in performance when comparing these results to the 50% AccB of a naive classifier, which would always predict all compounds to the majority class (non-toxic in this study). Furthermore, the FNN tends to produce more balanced TPR and TNR results compared to RF: a mean of 61.57% TPR and 76.22% TNR for the FNN opposed to 51.48% TPR and 85.02% TNR for RF. This observation is especially important when the task requires identifying potentially cytotoxic molecules in a highly imbalanced data set. Note that AccB, TPR and TNR are based on an automatically set cutoff yielding the maximum balanced accuracy on the respective validation split (mean of 0.17 for FNN and 0.07 for RF). The cutoff adaption is necessary because of the highly imbalanced nature of the underlying data set. This strategy is preferred over under-sampling in order to use as many data points as possible (see [58]).

**Table 2:** 10-fold nested cross-validation results (mean and standard deviation (std)) for the FNN and RF baseline models. Reported performance measures in percent (%) are balanced accuracy (AccB), true positive rate (TPR) and true negative rate (TNR). The best results on the test set are displayed in bold

		FNN			Random forest		
		AccB	TPR	TNR	AccB	TPR	TNR
Train	Mean	84.28	90.66	77.90	97.85	100.00	95.69
	Std	2.22	4.03	6.64	1.26	0.00	2.52
Val	Mean	70.13	63.94	76.32	68.72	52.35	85.09
	Std	1.30	6.92	6.82	1.71	6.96	5.70
Test	Mean	**68.89**	**61.57**	76.22	68.25	51.48	**85.02**
	Std	1.46	7.39	6.62	1.96	1.82	5.94

#### Comparison to other studies

Next, the CV results of the FNN and RF models trained on the FMP data are discussed in the context of three other recently presented models for cytotoxicity prediction [21, 23, 24], mainly using random forest models on freely available data (see Table 3). Note that results are only partly comparable between different studies since both data sets and methods may vary. Even in the case of same data, different splits can make comparison of methods difficult, as mentioned by Wu et al. [30].

Mervin et al. [21] trained a random forest model on publicly available NCBI BioAssay data, standardized using an in-house script. Molecules are considered cytotoxic if they have a pIC_50_ above 5.0 in the tested assay. Undersampling from millions of non-toxic molecules, the final public training data set contains a total of 14,880 molecules of which 3720 are labeled cytotoxic. With 25%, the share of toxic molecules is higher than in this study, but a similar weighting approach is used to balance the training data statistically. The external test data set consists of 988 molecules with an even higher share of 45% cytotoxic molecules [21, Table 8] and the model exhibits a balanced accuracy of 76.69%. Svensson et al. [24] trained a random forest model on extracted and standardized [45] molecules from PubChem, which were tested on a variety of cell lines and the cytotoxicity definition varied from one data set to the other. Their external data set consisted of 3295 molecules of which only 48 were labeled cytotoxic. Having a share of less than 1.5% is below the share of this study. Furthermore, they use conformal prediction models (CP) based on RF classifiers. The conformal prediction balanced accuracy of their model is 69.15%. However conformal prediction metrics do not necessarily translate to performance measured by metrics on pure model predictions. Banerjee et al. [23] report the highest balanced accuracy of 83.60% on their test data split. They extracted data from ChEMBL [59] and used cytotoxicity based on IC_50_ values at a concentration cutoff of $10\, \mu\hbox {M}$. The random forest classifier is trained on 5487 samples and evaluated on a test set of 610 samples, each containing one third of cytotoxic molecules [23, Table S1]. In the presented study, approximately seven times less toxic molecules were in the data set.

**Table 3:** Comparison of FNN and RF performance of this study with other existing models for cytotoxicity prediction (reported are mean CV results, noting that CV setup differ between methods). Balanced accuracy (AccB.), true positive rate (TPR) and true negative rate (TNR) are presented in percent (%). The last column describes the size of the test data, as well as the number and share of cytotoxic compounds. The best results are displayed in bold

					Test Set Size	
					Toxic	
Models	AccB	TPR	TNR	Total	Count	Percent
FNN (this work)	68.89	61.57	76.22	3437	160	4.6
RF (this work)	68.25	51.48	85.02			
RF, Mervin [21, Table 8, public]	76.69	56.90	**96.50**	988	445	45.0
CP/RF, Svensson [24, Table 5]	(69.15)	(73.80)	(64.50)	3295	48	1.5
RF, Banerjee [23, Table 2]	**83.60**	**93.00**	74.00	610	205	33.6

To conclude, Table 3 seems to suggest that models with more balanced data sets lead to better performance, as is illustrated with a 83.60% balanced accuracy from Banerjee et al. [23] and 76.69% from Mervin et al. [21]. However, as stated above, first, comparisons between the models should be made with care. Second, while having more balanced data sets may facilitate the modeling task, the question remains which resembles better the real live scenario. The results of the models trained on highly imbalanced data sets lie in the same range as shown with the FMP data and FNN as well as RF with a balanced accuracy of approximately 69% from this study and the RF-based CP model from Svensson et al. [24]. While Mervin et al. [21] obtain a TNP of 96.50%, the TPR is only 56.90%. In the FNN model used in this study, the TPR and TNR are more balanced, with a TNR of 76.22% and a TPR as high as 61.57%. This result may be more valuable in this context, since the main goal is to identify cytotoxic molecules. From an application point of view, correctly predicting cytotoxicity for novel molecules that would indeed later show toxic behavior (in in vitro or in vivo studies) may be more crucial, because these compounds could be excluded from further development.

#### FNN Production Run Results

After successful CV evaluation of the FNN model and comparison to a baseline RF as well as other published studies, a FNN was built for production run, showing a balanced accuracy of 70.73% on the test set. This model is used for the DTD in order to identify and highlight toxicophores in molecular structures.

**Table 4:** Model metrics in % at 0.17 cutoff yielding maximum balanced accuracy on the validation set (in bold) as well as another cutoff at 0.20 yielding higher TNR rates on the validation set (in bold)

	Cutoff = 0.17			Cutoff = 0.20		
	AccB	TPR	TNR	AccB	TPR	TNR
Train	85.76	92.50	79.02	86.43	89.53	83.32
Val	**69.46**	62.50	76.41	67.19	53.75	**80.62**
Test	70.73	63.12	78.33	69.53	56.88	82.18

**Fig. 4:**
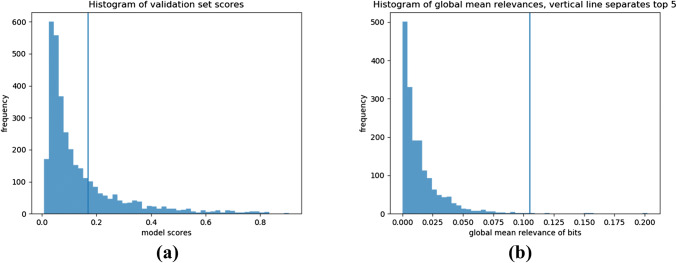
**a** Distribution of predicted scores for molecules from the validation set, which was used to calibrate the cutoff of 0.17 (indicated by the vertical line) of the model to classify compounds as cytotoxic. **b** Distribution of global mean relevances of set bits in decomposable compounds in the training and validation set, which were used to determine the five most important bits (indicated by the vertical line)

The cutoff value which yields the maximum balanced accuracy (69.46%) on the validation data is 0.17 (see Table 4 and Fig. 4a for the distribution of model scores corresponding to that specific cutoff). The TPR and TNR associated to that cutoff on the validation set are 62.50% and 76.41% respectively. Note that since the TPR and the TNR are directly related to a chosen cutoff, varying this cutoff value would immediately result in the change of these rates. Aiming towards a higher TPR or a higher TNR may depend on the research question at hand and the cutoff should be chosen accordingly. A cutoff of 0.20 would for example yield on the validation set a lower TPR of 53.75% but a higher TNR of 80.62% (see Table 4), and the same trend can be observed on the test set. Since the aim of this study is to reveal potential cytotoxic compounds which could then undergo further (experimental) testing, reaching a higher TPR is of more importance.

### Potential toxicophores

The current study aims to provide a visual structural interpretation of the model outcomes with the aim of identifying novel toxicophores. From the 30,929 molecules that are present in the training and validation set, a total of 1210 molecules are decomposable ($\sim$4%), which is in line with the share of cytotoxic molecules in the complete data set. As discussed in the “Identification of toxicopohores and visualization as cytotoxicity maps” section, relevance scores are obtained for each of the 2048 atom environments from these decomposable molecules. The workflow in Fig. 3 describes the process of going from decomposable molecules to global mean relevance scores per bit. Atom environments referring to high scoring bits generally contribute greatly to the predicted toxic value of the compound and thus represent potential toxicophores.

#### Identification of Potential Toxicophores Based on Most Important Bits

Note that for the analysis of the most important bits, global mean relevance scores were calculated per bit. These scores range from 0.0 to 0.2, and the distribution shows a drastic drop in values indicating that only few bits have a high impact (see Fig. 4b). In the following, the $k=5$ bits with the highest scores are selected for further analysis. Note that with increasing values of *k*, more often several of these bits appear together in one molecule and overlap. Thus, the portion of the molecule that is covered by these bits, which likely contribute to cytotoxicity, becomes larger and closer to a full scaffold. In this case study, selecting the five highest relevance scores seems appropriate to reveal meaningful substructures. Table 5 displays these bits in decreasing order with respect to the global mean relevances as well as the predictions (TP and TN counts) given by the FNN model. On the training and validation set, the molecules that contain at least one of these bits are correctly predicted cytototoxic by the model 85% of the time. If the counts from bit 85 are removed, this number increases to over 90%. Similar findings can be assessed on the test set: the model yields 69% and 75% correctly predicted values, including and excluding bit 85, respectively. This observation indicates two facts: First, the results of the DTD method are meaningful and useful in assessing the cytotoxicity of compounds. Novel molecules containing these bits should be treated with special attention in future laboratory experiments. Second, bit 85 seems to be an outlier which will be discussed later in greater details.

**Table 5:**
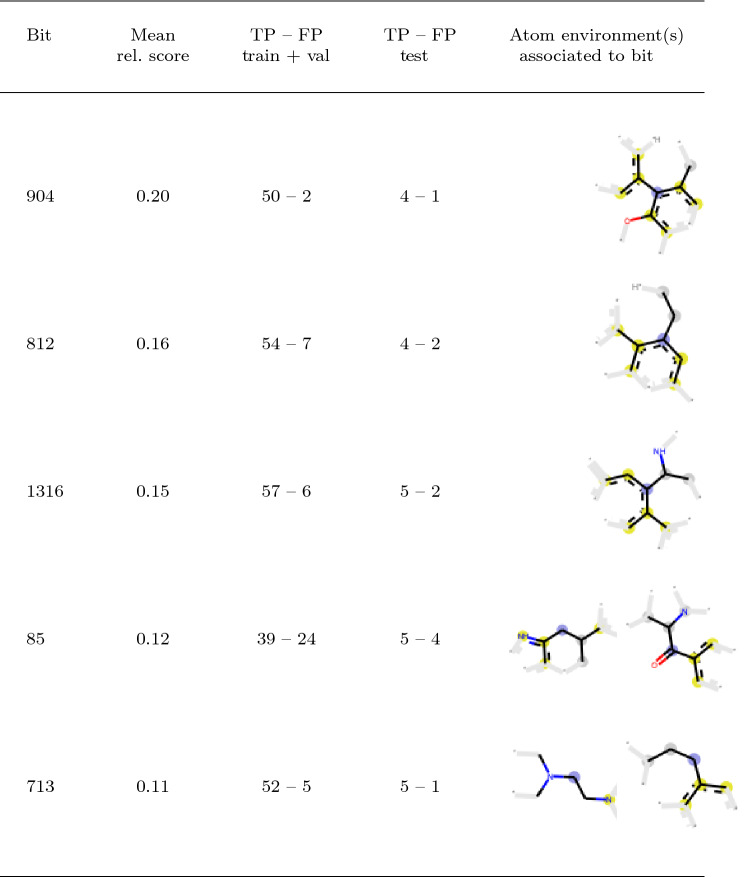
Bits with the five highest global mean relevance scores (rel. score) are shown in decreasing order, as well as the predictions (TP and FP counts) given by the FNN model on both the training and validation sets (train+val) and on the test set for molecules that contain these bits. The last column shows the 2D image of atom environments associated to the Morgan fingerprint bit in the test set (two images to exemplify bit collisions), where the blue, yellow and gray circles represent central, aromatic and aliphatic ring atoms, respectively

**Fig. 5:**
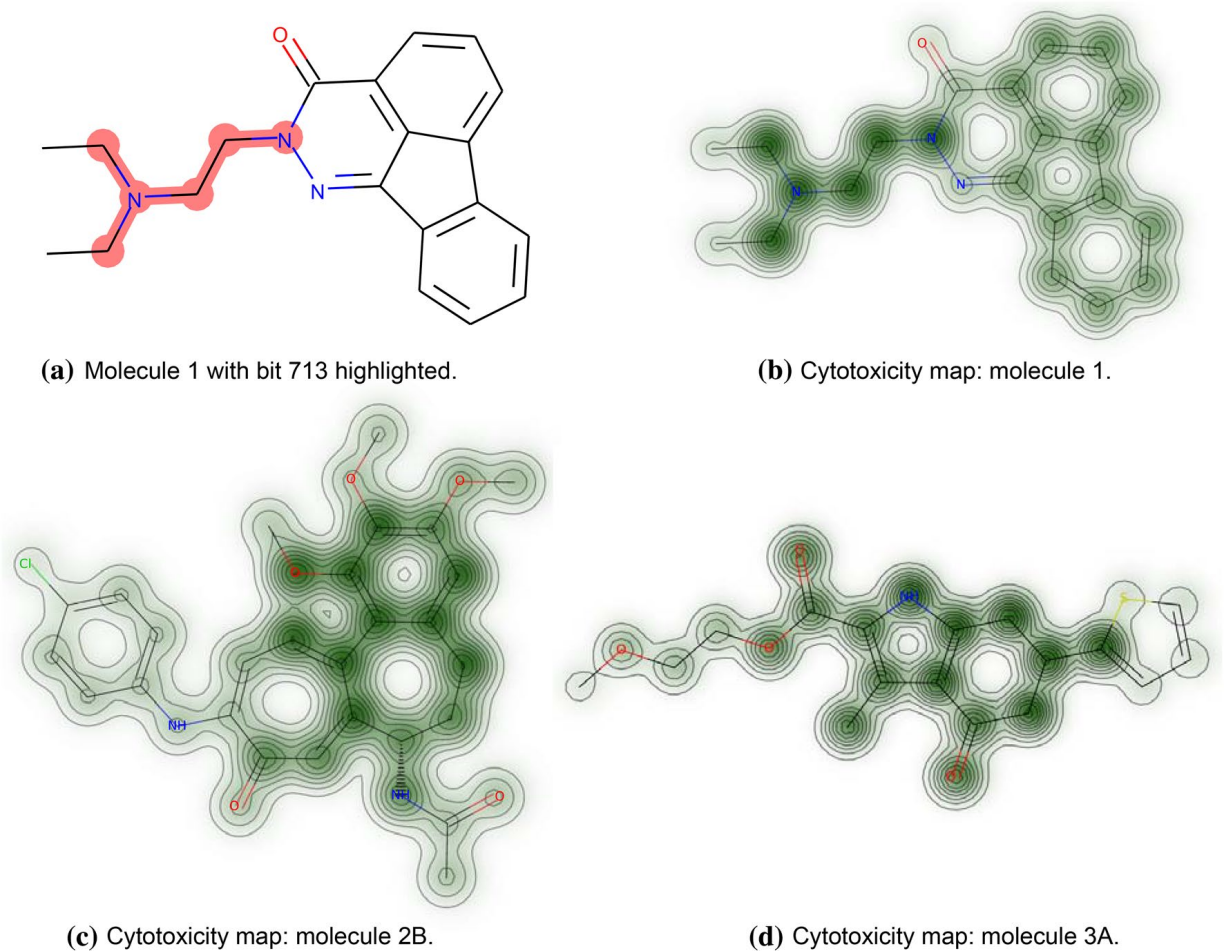
The figure shows three compounds from the test set, namely molecule 1, molecule 2B and molecule 3A, that were correctly labeled cytotoxic by the FNN model. **a** highlights bit 713 in red in molecule 1. **b**–**d** illustrate the cytotoxicity maps for these molecules. The atomic weights are computed using the approach discussed in the “Identification of toxicopohores and visualization as cytotoxicity maps” section. The higher the value of the respective global mean relevance, the darker the green coloring

In the test set, 17 molecules contain at least one of these top five atom environments (see Fig. S1 in the Supplementary Material, bits highlighted in red). For example, test molecule 1, an indenophtalazinone derivative, was correctly labeled cytotoxic by the FNN model and contains bit 713 (see Fig. 5). To verify this prediction, the eMolTox tool developed by Ji et al. [60], an in silico drug safety analysis system, was queried. The authors constructed Mondrian conformal prediction models for 174 toxicology-related in vitro and in vivo experimental data sets. eMolTox predicts the compound with high confidence as potentially being genotoxic, interacting with the CNS, and/or with the liver. Most interesting are two similar compounds that exist in the underlying database which were tested active in the context of genotoxicity (i.e. the drug flurazepam, ChEMBL968 in the ChEMBL data base [59]) and liver damage (amonafide, Phase III, ChEMBL428676). While the annelated scaffold systems of these active molecules, such as the benzodiazepine scaffold from flurazepam differ from the compound in this study, they also contain the tertiary substituted ethylendiamine corresponding to bit 713 in molecule 1. Moreover, eMolTox offers the detection and highlighting of toxic substructures in each query molecule, based on a list of structural alerts collected from literature (see Table S2 in Ji et al. [60]). For the query molecule, several structural alerts are identified. Among them, the tertiary amine is highlighted being potentially involved in covalent DNA binding. The toxicophore identified here seems to contain but extend the known structural alert to a larger moiety that is potentially involved in cytotoxicity. Figure 5 illustrates the cytotoxicity map for the considered molecule. The atom environment associated to bit 713 stands out compared to the other substructures in the molecule and therefore may be designated as a toxicophore. Furthermore, the right part of the fused ring system also shows some intensity (relevance) and actually describes a part of the molecule that was also highlighted by eMolTox’s structural alerts and annotated as potentially kidney toxic or hepatoxic.

**Fig. 6:**
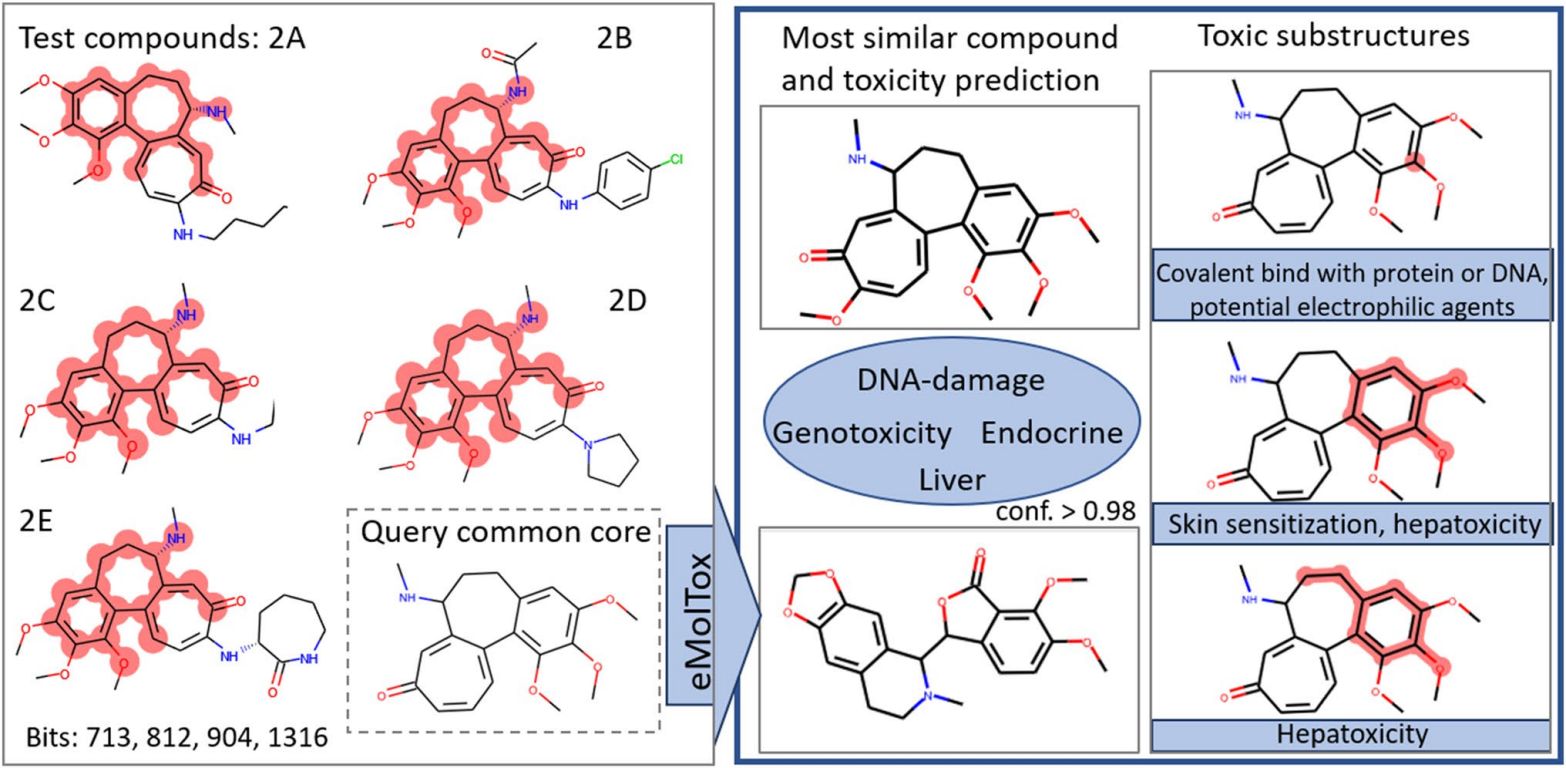
Schematic description of analysis: On the left, molecules 2A-2E from the test set are shown together with the relevant bits highlighted in red. The common core of these five molecules is used as query for the eMolTox server and the results of eMolTox are summarized on the right, with predicted toxic endpoints in blue

Additionally, in five molecules of the test set (2A-2E in Fig. 6, see also Fig. S1 in the Supplementary Material) four of the five most relevant bits (namely bits 713, 812, 904, 1316) appear together and form a potential toxicophore which covers a larger 6,7-dihydrobenzo[a]heptalen-9(5H)-one core structure including methoxy and amino substituents. This combined substructure is present in five compounds from the test set of which four are indeed experimentally labeled cytotoxic (molecules 2A to 2E in Fig. 6, left) and the FNN predicts them as toxic with a high mean probability of 0.89 (see Table S2 in Supplementary Material). This assumption is supported by the cytotoxicity map exemplified for test molecule 2B (see Fig. 5c).

Using the eMolTox tool, a toxicity prediction for the visually determined maximum common substructure of these five compounds was performed (see Fig. 6). The most similar active compound in the eMolTox data set to the queried common core is the known drug demecolcine (ChEMBL312862), a colchicine derivative, which is used in chemotherapy and shows cytotoxic activity. In accordance with being predicted cytotoxic in this study, the queried common substructure is predicted by eMolTox to further cause DNA damage, genotoxicity, as well as interacting with the liver and endocrine system (see Fig. 6, right). Furthermore, eMolTox identified the following toxic alerts: covalent binding to proteins or DNA (because of potential electrophilic reactivity), as well as skin sensitization and/or hepatoxicity (the latter two caused by catechol or catecholdimethyl ethers or p-alkoxy aromatic ethers). The identified 4-bit substructure in this study extends the alerts and suggests a larger substructural entity, namely the 6,7-dihydrobenzo[a]heptalen-9(5H)-one core structure bearing methoxy and amino substituents, being involved in cytotoxicity (see Fig. 6).

As described above, bit 85 was identified as one of the five bits with the highest global mean relevance for cytotoxicity and thus, a potential toxicophore. Surprisingly, in the training and validation set, only 39 out of the 63 decomposable molecules containing this bit were experimentally tested as cytotoxic (61.9%). In contrast, high precision (TP/(TP+FP)) ranging between 88.5% and 96.2% were achieved for the decomposable molecules containing one of the other four bits (see Table 5). Also, 4 out of 9 decomposable molecules in the test set containing bit 85 are falsely predicted as toxic. Therefore, bit 85 was further analysed uncovering two interesting aspects: First, five different atom environments are mapped to bit 85, of which the two most common ones (72% and 10%, named bit 85_t1 and 85_t2 in the following) are depicted in Table 5 and are present in molecules 3A to 3G and in molecule 4 of the test set, respectively (Fig. S1 in the Supplementary Material). This behavior is known as bit collision when working with folded molecular fingerprints, as mentioned in the “Data” section. Folding is a compromise between accuracy and performance since unfolded fingerprints can become enormously long. In this study, the unfolded fingerprints could already be reduced to a size of 14,245 bits by introducing a filtering step, but are afterwards folded to 2048 bits, as described in the “Data” section. Considering the 63 decomposable molecules containing an atom environment that is mapped to bit 85, 52 cases represent type 85_t1, the remaining 11 type 85_t2 (see Table 5). All molecules from the latter group were indeed experimentally tested toxic (similar to molecule 4). In contrast, almost half of the 52 molecules of the former group (similar to molecules 3A to 3G) were experimentally tested non-toxic (FPs). This indicates that the model could be improved by reducing such bit overlap. Note that these collisions seem to be less problematic in the case of bit 713. Most of the decomposable molecules in the training set which contain bit 713, with different associated atom environments (as shown in Table 5), do indeed belong to the toxic class. Second, the low precision for compounds containing bit 85 points to the fact that this class of molecules might be challenging for the algorithm. While having a common 1,5,6,7-tetrahydro-4H-indol-4-one core, the toxicity of the compounds seems to depend on the peripheral substitution and the functionalization. This points to the concept of activity cliffs, which are a challenge for many predictive modelling approaches [61]. While the FNN generates many FPs for the decomposable molecules of this compound class, the algorithm nevertheless predicts the TPs (3A in Fig. 5d, 3C, 3D and 3G) with higher mean probability than the FPs (3B, 3E, 3F and 3H), 0.77 vs. 0.64, respectively (see Table S2 in Supplementary Material).

Note that molecule 5 (which contains bit 1316) and molecule 6 (which contains bit 812) are wrongly predicted as cytotoxic by the FNN. The most relevant bits they contain refer to bit collision and are different from the major bit types shown in Table 5. Furthermore, the predicted scores are slightly lower than for the TPs mentioned above, i.e. 0.59 for molecule 5 and 0.69 for molecule 6 (see Table S2 in Supplementary Material).

These observations highlight the value of the DTD method during model setup and evaluation. Using the features learned by the algorithm and mapping the scores back to the structure, shortcoming of the model can be pinpointed and actions could be taken such as enlarging the fingerprint length to minimize bit collision, or to investigate in more detail specific difficult compound classes in the data set.

#### Cytotoxicity maps and comparison to other methods

Besides the identification of such novel toxicophores, the DTD relevance scores of all atom environments in a molecule can be depicted to produce a cytotoxicity map of the molecule (adapted from the similarity maps [40] as also used by Preuer et al. [35, Fig. 4]). Thus, the decomposition of a single molecule is presented entirely which allows easy interpretation of the results, as shown in Fig. 5b–d. In this study, the DTD approach is used to select relevant bits to be able to interpret what the model learned. Furthermore, this provides a data-driven approach to identify novel toxicophores.

Other approaches exist that try to unleash the black box in ML, for example, Mayr et al. [33] compare the neurons in the network to predefined toxicophores. Sheridan [34] uses a leave-one-feature-out approach on many different modeling settings in order to identify feature importance. Relevances are assigned based on the difference between model scores with a particular feature being present and absent. Recently, Manica et al. [36] published an attention-based neural network architecture to predict IC_50_ values for known drugs using RNA and SMILES data. The attention vector is calculated from the latent representations and is used to identify the most relevant latent features [62] in the SMILES encoding. Closest to the study presented here is the work by Preuer et al. [35]. In spite of technical details such as model architecture, data set, and input featurization, both studies try to understand the toxic mechanism using deep learning. However, not only are the endpoints that are considered different, but the problem is tackled from different angles. The study by Preuer et al. [35] investigates, among other, the role of units in hidden layers as pharmacophore detectors and the issue of bit collision is not addressed. Moreover the method used to investigate the interpretability of neural networks, the so-called Integrated Gradients Method, is different from the Deep Taylor Decomposition as presented in this study. The Integrated Gradients Method, as the name suggests, integrates all the gradients that lie on the path between an input *x* and a predefined baseline $x'$ to obtain a score for each dimension of the input. The integration is numerically approximated by a sum, where the number of steps is predetermined. Obtaining an accurate approximation of this integral requires many time steps (1000 in the study by Preuer et al. [35]). When comparing the DTD method to Integrated Gradients, DTD is computationally more efficient as only one backpropagation is needed to assign relevances in comparison to 1000 time steps for a single decomposition in [35]. Both Integrated Gradients and leave-one-feature-out are model agnostic and straightforward to apply, but in contrast the DTD is very intuitive and consistent.

## Conclusion

In this study, a deep learning approach to predict the cytotoxicity of compounds is presented using a highly consistent data set of over 34,000 compounds provided by the FMP. Note that the data was composed as screening data set, thus not focusing on cytotoxicity, which led to a low share of cytotoxic molecules. Most importantly, a procedure is introduced to make deep learning models more interpretable. In this way, the Deep Taylor Decomposition is used to identify toxicophores in a molecule from a fully-connected feedforward neural network by mapping relevance scores back to atom environments.

The results of the experiments show that the model is competitive with the current literature given data sets with similar share of toxic and non-toxic molecules. The best balanced accuracy on the test set which the FNN model reached is as high as 70.73% which is significantly better than naive classification at 50% and the FNN model yielded more balanced results than the baseline RF model. Moreover, using the DTD method, atom environments could be identified which are likely to be involved in cytotoxic behavior of the compounds. As example, the five atom environments with the highest global mean relevance scores were identified and discussed in this study. Molecules in the test set containing these bits were mostly correctly predicted cytotoxic by the FNN model. These findings are coherent with the current literature and especially some of the identified substructures extend the known list of structural alerts. Furthermore, cytotoxicity maps are generated that highlight the contribution of each individual bit, which allow chemists to identify, from these plots, their own relevant toxicophores in newly synthesized compounds.

One aspect that should be considered carefully when applying the approach developed in this study to new molecules is to verify that the compounds are in the scope of the model. For more details on the concept of defining the applicability domain, please refer to Hanser et al. [63]. Generalization to the entire chemical space may be difficult when training any ML model on a static data set. Furthermore, regarding the input features of the model, a noticeable limitation of fingerprints is bit collision which may be ambiguous when trying to identify substructures likely to produce toxic compounds. Using longer fingerprint vectors may help prevent bit collision. An alternative would be to choose a different molecular encoding, such as the SMILES representation as in [64], or a learned representation as developed by Winter et al. [65].

Concluding, the study presents a novel way of interpreting the outcome of the FNN model to help understand what the model learned in the context of molecular toxicity. While most toxicophores are selected by humans, the relevance scores together with the cytotoxicity maps are a technique that identifies these substructures in a data-driven fashion. Spotting such substructures at an early stage of drug design can be highly beneficial for pharmaceutical research to reduce costly and timely laboratory experiments.

## Electronic supplementary material

Below is the link to the electronic supplementary material.

Supplementary file1 (PDF 91 kb)

## Footnotes

1 Hoechst 33342 is a cell-permeable minor groove-binding DNA stain, which starts to fluoresce bright-blue upon DNA binding. Stained nuclei are then easily distinguishable from background using fluorescence excitation in the UV range.
