# Supplementary material for: Revealing cytotoxic substructures in molecules using deep learning
Source: J Comput Aided Mol Des. 2020 Apr 16;34(7):731–46. doi: 10.1007/s10822-020-00310-4 (PMC7292813; doi:10.1007/s10822-020-00310-4)
Supplement: Supplementary file 1 — Supplementary file1 (PDF 91 kb) [file 10822_2020_310_MOESM1_ESM.pdf]

REVEALING CYTOTOXIC SUBSTRUCTURES IN MOLECULES USING DEEP LEARNING

Henry E. Webel, Talia B. Kimber, Silke Radetzki,
Martin Neuenschwander, Marc Nazaré, Andrea Volkamer

Supplementary Material

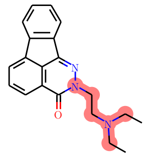
test mol 1, TP, bits: 713

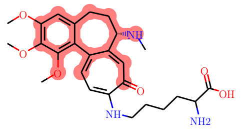
test mol 2A, FP, bits: 904 1316 812 713

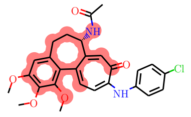
test mol 2B, TP, bits: 904 1316 812 713

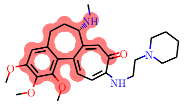
test mol 2C, TP, bits: 904 1316 812 713

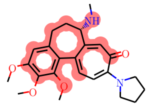
test mol 2D, TP, bits: 904 1316 812 713

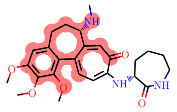
test mol 2E, TP, bits: 904 1316 812 713

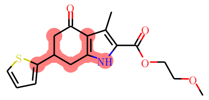
test mol 3A, TP, bits: 85

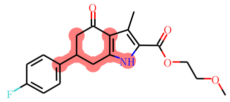
test mol 3B, FP, bits: 85

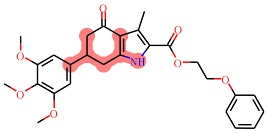
test mol 3C, TP, bits: 85

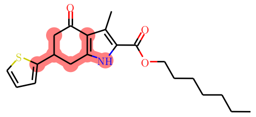
test mol 3D, TP, bits: 85

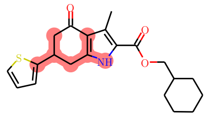
test mol 3E, FP, bits: 85

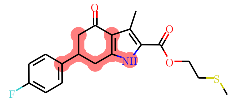
test mol 3F, FP, bits: 85

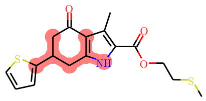
test mol 3G, TP, bits: 85

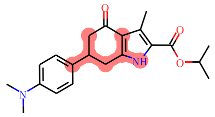
test mol 3H, FP, bits: 85

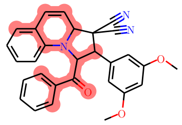
test mol 4, TP, bits: 1316 85

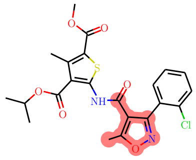
test mol 5, FP, bits: 1316

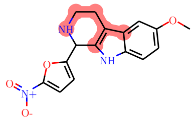
test mol 6, FP, bits: 812

Figure S1. The Deep Taylor Decomposition was used on a feed-forward neural network to assign a relevance score to each atom environment, which can be used to identify potential toxicophores. The five highest scores were selected and are associated to bits 85, 713, 812, 904 and 1316. The figure shows molecules in the test set which contain at least one of the five atom environments, highlighted in red. The label for each molecule specifies its name, weather it was correctly predicted cytotoxic (TP) by the model or not (FP: False Positive) and lastly the bit(s) it contains.

Table S2. The table shows the IDs of the decomposable molecules in the test set sorted by decreasing order with respect to the FNN model prediction probability, the true value experimentally determined (1: toxic, 0: non-toxic) and the value predicted by the model (TP: true positive, FP: false positive).

Molecule ID	FNN Score	True Value	Predicted Value
2E	0.91	1	TP
2C	0.90	1	TP
2B	0.89	1	TP
2D	0.89	1	TP
2A	0.86	0	FP
4	0.83	1	TP
3C	0.79	1	TP
3D	0.78	1	TP
3A	0.78	1	TP
1	0.75	1	TP
3G	0.72	1	TP
6	0.69	0	FP
3B	0.68	0	FP
3H	0.67	0	FP
3E	0.61	0	FP
3F	0.61	0	FP
5	0.58	0	FP
